# Predicting the development of T1D and identifying its Key Performance Indicators in children; a case-control study in Saudi Arabia

**DOI:** 10.1371/journal.pone.0282426

**Published:** 2023-03-01

**Authors:** Ahood Alazwari, Alice Johnstone, Laleh Tafakori, Mali Abdollahian, Ahmed M. AlEidan, Khalid Alfuhigi, Mazen M. Alghofialy, Abdulhameed A. Albunyan, Hawra Al Abbad, Maryam H. AlEssa, Abdulaziz K. H. Alareefy, Mohammad A. Alshamrani

**Affiliations:** 1 School of Science, RMIT University, Melbourne, Victoria, Australia; 2 School of Science, Al-Baha University, Al-Baha, Saudi Arabia; 3 King Fahad Medical City (KFMC), Riyadh, Saudi Arabia; 4 Maternal and Children Hospital, Al-Ahsa, Saudi Arabia; King Abdulaziz University, SAUDI ARABIA

## Abstract

The increasing incidence of type 1 diabetes (T1D) in children is a growing global concern. It is known that genetic and environmental factors contribute to childhood T1D. An optimal model to predict the development of T1D in children using Key Performance Indicators (KPIs) would aid medical practitioners in developing intervention plans. This paper for the first time has built a model to predict the risk of developing T1D and identify its significant KPIs in children aged (0-14) in Saudi Arabia. Machine learning methods, namely Logistic Regression, Random Forest, Support Vector Machine, Naive Bayes, and Artificial Neural Network have been utilised and compared for their relative performance. Analyses were performed in a population-based case-control study from three Saudi Arabian regions. The dataset (n = 1,142) contained demographic and socioeconomic status, genetic and disease history, nutrition history, obstetric history, and maternal characteristics. The comparison between case and control groups showed that most children (cases = 68% and controls = 88%) are from urban areas, 69% (cases) and 66% (control) were delivered after a full-term pregnancy and 31% of cases group were delivered by caesarean, which was higher than the controls (*χ*^2^ = 4.12, P-value = 0.042). Models were built using all available environmental and family history factors. The efficacy of models was evaluated using Area Under the Curve, Sensitivity, F Score and Precision. Full logistic regression outperformed other models with Accuracy = 0.77, Sensitivity, F Score and Precision of 0.70, and AUC = 0.83. The most significant KPIs were early exposure to cow’s milk (OR = 2.92, P = 0.000), birth weight >4 Kg (OR = 3.11, P = 0.007), residency(rural) (OR = 3.74, P = 0.000), family history (first and second degree), and maternal age >25 years. The results presented here can assist healthcare providers in collecting and monitoring influential KPIs and developing intervention strategies to reduce the childhood T1D incidence rate in Saudi Arabia.

## Introduction

Type 1 diabetes (T1D), commonly known as insulin-dependent diabetes, is a metabolic illness caused by an autoimmune reaction that attacks the pancreas’ insulin-producing beta cells, resulting in severe insulin deficiency and accompanying hyperglycemia. A deficiency of insulin production is characteristic of the disease, and insulin injections must be administered continuously for the rest of one’s life for survival [1]. Diabetes complications can result in a variety of long-term health issues, such as nerve and blood vessel damage, hospitalization, and death [2, 3]. Additionally, T1D can cause blindness, due to retinopathy [3], and kidney failure [4]. Chronic diseases like diabetes can disrupt physiology, affecting linear growth, pubertal development [5], and cause serious dermatological complications [6].

T1D is likely to be initiated by as yet unidentified environmental variables in genetically susceptible individuals, with the major genetic contribution identified within the HLA complex, specifically HLA class II [7]. Despite recent advances in the understanding of the disease’s genetics and immunology, its incidence continues to rise by 3–4% per year worldwide [8]. T1D is a key primary target for prevention due to its growing incidence, severe morbidity and mortality, and substantial health care costs [8]. According to the International Diabetes Federation (IDF), Atlas’ 10th edition (2022), there are 651,700 children under the age of 15 years globally with T1D [9]. Additionally, it is predicted that approximately 108,300 children under the age of 15 will be diagnosed with T1D each year, rising to 128,900 when the age range is extended to 20 years [9]. Saudi Arabia has a high incidence rate of new T1D cases in children (<15 year) each year (31.4 cases/100,000 children) and the number of new T1D cases among children and adolescents is estimated to be 3,800 annually [9].

Better knowledge of the development of T1D in Saudi Arabian children is urgently needed to inform monitoring practice in order to prevent complications and address the incidence of T1D, as early intervention may have a higher success rate in preventing the beginning of dysglycemia and slow the loss of functioning islet cells.

### Review of literature on risk factors of T1D in children

The etiology of T1D is uncertain, environmental triggers and viruses are believed to activate the autoimmune process, resulting in pancreatic B-cell destruction and T1D [10]. Although the involvement of environmental and genetic influences on the development of T1D has been recognised for over 40 years [11], research into the potential risk factors for T1D is ongoing [12, 13]. Demographic and socioeconomic status, genetic, obstetric history, maternal characteristics and nutritional history have all been associated with T1D onset [12–14], although the relative contribution of each factor shows variation in the literature as outlined below.

### Demographic and socioeconomic status

The research on the demographic profile has investigated and compared T1D incidence in urban and rural residents. An Australian study reported that the incidence of T1D in children in urban areas increased by an average of 2.5% a year (95% CI [1.2, 3.8]) compared with 5.0% a year (95% CI [2.4, 7.6]) in the non-urban area over the period from 1985 to 2002 [15]. This difference in incidence-rate trends was not statistically significant over the whole study period. However, the rate of increase in incidence in non-urban areas was significantly higher than that in urban areas when this study was limited to the last 10 years [15]. In addition, a study conducted in Taiwan found that there was an association between the incidence of T1D and living in an urban area [16]. However, studies conducted in Finland and Scotland [17, 18] indicated that urban areas were linked to lower incidence rates compared to rural areas. A recent study conducted in Germany also found that the incidence of T1D was higher in rural areas compared with urban areas [19].

The apparent difference in patterns of incidence between European countries and those of Asia and Australia could be due to the socioeconomic differences between these environments. A study conducted in Australia indicated that T1D incidence is more likely in families with a higher socioeconomic status [15]. This is supported by the findings of a study of European countries that showed T1D rates are most closely correlated with markers of country prosperity such as gross domestic product and low infant mortality [20]. T1D incidence was also shown to be the lowest in areas with the most material deprivation in the United Kingdom (Northern Ireland [21], Yorkshire [22], and Scotland [18]).

### Genetic (family history)

It is well established that there is a genetic component that increases the risk of T1D in children. It is shown that children of mothers with T1D have a greater risk of developing T1D compared with offspring of non-diabetic mothers [23]. In [24], the authors showed that the risk of T1D for children whose fathers are affected by the disease is 11 times higher than in the control group [14]. While the risk for children whose brothers are affected by the disease is 20 times higher compared to the control group [14]. [7] presents a review and comparison of the genetic determinants of T1D in various populations. They have concluded that the worldwide variation in incidence is at least partially determined by differences in genetic risk factors. Authors in [24] have shown that children with the HLA-risk genotype along with a family history of T1D have more than a 1 in 5 risk for developing islet autoantibodies during childhood. The authors in [7] have found over 40 loci were associated with the risk of T1D.

### Nutritional history

When considering nutrition of the child, a recent review study reported that early exposure to cow’s milk could lead to the development of T1D in children [25]. Other studies reported that short breastfeeding periods (2–4 months) and early cow’s milk exposure (before 4 months) might increase susceptibility [26]. The absence of breastfeeding was found strongly associated with T1D [27]. Another study [28] has explored the associations between early solid food feeding and the risk of T1D. The authors found that early (<4 months) and late (>6 months) introduction of solid foods was associated with increased risk of T1D. Furthermore, studies from Saudi Arabia [29, 30] and other countries such as Italy [31] found that vitamin D deficiency may be linked to the occurrence of childhood T1D.

### Obstetric history

Several studies have examined associations between obstetric factors and T1D such as birth delivery method, gestational age, birth weight, and birth order. Birth delivery method was linked to the development of T1D in children [32–35]. It is well documented that children born by Caesarean section have a higher propensity to develop T1D compared to children born by normal delivery [32–35]. For gestational age, very preterm (<33 weeks) [35, 36] or post-term (>40 weeks) [35] were linked to a reduced risk of the onset of T1D in children. However, pre-term (33–36 weeks) and early term (37–38 weeks) were linked to an increased risk of T1D [35–37].

An association between a higher birth-weight (of the child) and the risk of developing T1D was found in [35, 38]. Children weighing either 3.5 kilograms (kg) to 4.0 kg or heavier than 4.0 kg at birth had an average increase of 6% and 10%, respectively in their risk of diabetes [38].

Birth order may also contribute to the risk of developing T1D. A previous study indicated that the risk in firstborn children was highest and gradually declined with higher birth order after adjustment for the effects of maternal age at childbirth and child sex [39]. This is also supported by [40] where a higher birth order was associated with a substantial reduction in the risk of T1D comparing firstborn birth order with three or more.

### Maternal characteristics

Maternal health may play an important role in T1D. In a comparison of maternal age of greater than 35 with maternal age of less than 25, the occurrence of T1D in the older maternal age group increased substantially [40]. This was also confirmed by [39], who showed an increased chance of having T1D for children with a mother of 45 years or more.

Previous studies reported that maternal diabetes (T1D, T2D, or gestational diabetes) is associated with an increased risk in children with T1D [23, 37]. Other maternal health concerns such as maternal asthma are associated with an increased risk of T1D in children [37]. Furthermore, pre-eclampsia was considered a significant risk factor for the development of T1D in children [41]. However, a meta-analysis study showed that children born to mothers with pre-eclampsia had on average 10% increase in their risk of developing T1D but this association was not significant [42].

### The use of preventative studies to improve T1D clinical outcomes

The possibility of preventing, delaying, or reducing complications of T1D diabetes in children is an important area of investigation [3, 43, 44]. In the preventative studies [45, 46] it was demonstrated that the elimination of cow’s milk proteins in infant formula (in the Finnish TRIGR pilot study [45]) and the elimination of bovine insulin in infant formula (in the FINDIA study [46]) both decreased the production of islet autoantibodies. Further studies have investigated methods for predicting T1D. Early exposure to respiratory infections has been linked to an increased probability of autoantibody seroconversion in children with a T1D family history [47]. This was observed by continuously monitoring islet autoantibodies over their first three years of life [47]. The risk of T1D has also been predicted using longitudinal autoantibody measures in families with a first-degree relative with T1D [48], in the general populations [49], or in individuals who have been identified as at risk [48, 50, 51]. In addition, genetic markers and genetic risk scores were used to detect islet autoantibodies in children with high-risk HLA genotypes [52, 53]. A composite risk score model that was constructed for high-risk children that included clinical, genetic, and immunological variables demonstrated a significantly improved T1D prediction compared to autoantibodies alone [54]. The benefits shown by the above research could be enhanced further by including more of the risk factors of T1D.

### Machine learning to predict T1D development in children

Creating predictive model with the large number of risk factors requires a sophisticated approach. Machine learning is becoming a popular and important approach in the field of medical research due to its capability of modeling complex linear and nonlinear relationships. Researchers in T1D have started to use Machine learning methods [55–58]. In [55], the authors utilised Random Forest (RF), Support Vector Machines (SVM), and Generalized Linear Model (glmnet) with data from the intestinal microbiota of infants to search for species that influence the development of T1D. In addition, the authors in [56] used Naïve Bayes classifier to determine if a combination of genetic, immunologic, and metabolic features, measured at infancy, can be used to predict the likelihood that a child will develop T1D by age 6 years. In another study [57], Naïve Bayes, Decision Tree, Support Vector Machine SVM, and Random Forest were used to predict diabetes in children using glucose, blood pressure, skin thickness, insulin, BMI, age, and family history of diabetes. In [58], the authors used Random Forest and logistic regression to evaluate the association between the onset of T1D in a child and the mother’s vaginal bacteriome and mycobiome. However, beyond the above research, there is an opportunity for further application of machine learning methods to develop predictive models of T1D.

### Motivation and the objective of this study

The existing research such as those conducted in Australia, Sweden, and Finland do not capture the diversity, ethnicity, and culture of the population in Saudi Arabia [59, 60]. It is reported [61] that there is a high rate of consanguinity in Arabic countries which could lead to increased homozygosity in the (HLA) haplotypes and non-HLA genes. This may alter the susceptibility to T1D. In addition, Saudi Arabia has an increasing incidence rate of T1D in children, ranking 7th in the world for the highest number of children with T1D and 5th in the world for the T1D incidence rate [9]. Despite the remarkably increased incidence of childhood T1D in Saudi Arabia, there is a lack of comprehensive research on T1D in Saudi children when compared to developed countries [62–64]. Existing T1D research in Saudi Arabia used small sample sizes, a single center, or a single city or region of the country, or limited number of factors [62, 64, 65]. Improvements are being made with a recent study conducted over three different regions in Saudi Arabia exploring age at onset of T1D in children [64], however this can be further enriched with the incorporation of control data as in this study. This study aims to fill the gap by creating predictive models of T1D and identifying the KPIs of T1D in children using data from Saudi Arabia. The proposed work differs from previous research as we have considered:

1. Incorporating the multiple Key factors identified above to create a more robust predictive T1D model in children using a wider range of environmental and family history factors.
2. Both statistical and modern machine learning models have been explored to determine the best model and identify the KPIs of T1D.
3. Using a reasonable sample size of both cases and matched controls (birth year, gender, and location) to create a more robust and clinically relevant model by controlling confounders. This is supported by using a larger and more comprehensive representation of the country’s diverse population.

Having a clinically relevant predictive model will contribute to better understanding and identifying the requirements to begin the development of a clinically relevant tool. This tool can then be used to develop intervention strategies to reduce the incidence rate of Childhood T1D in Saudi Arabia. The research presented here will improve public health in Saudi Arabia and contribute to filling the gap in T1D research for diverse populations.

## Data and methods

### Population and sample size

De-identified data for 1,142 individuals were collected based on a case-control study conducted in three cities (Al-ahsa, Jeddah, and Riyadh) located in the highest populated regions of Saudi Arabia; Eastern, Western, and Central to address the aim of this work. Ethical approval was granted by the RMIT University Human Research Ethics committee in Australia and the Research Ethics Committee of the Ministry of Health in Saudi Arabia. This was a retrospective study of medical records, with additional supporting demographic information collected through a survey of the parents of each child included in the cohort. The need for informed consent was waived by the ethics committee to collect existing data from medical records. Informed consent was obtained for the additional information collected via survey for their residency, income status, and nutritional history. All data were collected and reviewed by trained medical professionals and then were fully anonymised before analysis. Cases were children <15 years with a confirmed diagnosis of T1D between 2010 and 2020. Controls were children <15 years without any clinical indicators of T1D who attended the same hospital. All children with diabetes were matched with at least two control children with the same year of birth, same sex, and from the same city. In this study, the sample sizes were total: 1,142, cases: 377 and controls: 765. An *a priori* power calculation using the epiR package in R, indicated that a total sample size of 1,086 would be sufficient at 80% power with a 95% confidence level to detect an OR above 1.5. This was determined with a 1:2 case to control ratio and a prevalence of control exposure set to 24%, as reported in previous literature [66–68].

### The Key Performance Indicators (KPIs) considered

A structured questionnaire was used for collection of data from the medical records and parents of both case and control samples. De-identified data were collected on socio-demographic, potential genetic and environmental factors identified through the literature review. The collected demographic data included city where they live, gender, and birth year. Socioeconomic Status: residency and family’s income levels factors. Genetic: consanguinity marriage and having a family history of T1D (First degree and second degree). Environmental: nutrition history, and solid food, obstetric History: birth delivery mode, gestational age, weight at birth (Kg) and birth order. Maternal characterise at child birth: maternal age at child’s birth, gestational diabetes, maternal asthma and Pre-eclampsia. The pregnancy weeks used by the World Health Organization (WHO) and the American College of Obstetricians and Gynaecologists (ACOG) [69, 70] were used to categorise pregnancy length (in weeks) as: <33 (very pre-term), 33–36 (pre-term), 37–38 (early-term), 39–40 (full-term), 41 (late-term), and >42 (post-term). The child’s weight at birth was categorised into 4 categories using WHO and ACOG definitions; Birth weight (<2.5 kg), Birth weight (2.5–3.0 kg), Birth weight (3.5–4.0 kg), and Birth weight (>4 kg) [71, 72]. Also, maternal age at child birth were classified to 3 classes based on the previous studies [40]; maternal Age at child’s birth: (<25 years), maternal age (25–35 years), and maternal age (>35 years). Income was collected as a selection from the following 5 categories: High income: >12000 Saudi Riyals, Upper-middle income:(>9000–12000) Saudi Riyals, Middle income: (>6000–9000) Saudi Riyals, Lower-middle income: (>3000–6000) Saudi Riyals, and Low income: ≤ 3000 Saudi Riyals.

### Logistic regression and ML models

Understanding the wide range of the potential KPIs of T1D could ease diagnosis, provide adequate classification and relative importance and allow for cost-effective disease management. Therefore, the first phase in effective intervention is to identify such KPIs associated with T1D. Majority of conducted T1D studies in children have used a single approach to identify KPIs whereas using different and comparable approaches can enhance the ability to find the best fit for the data and hence may more accurately provide the significant KPIs. Machine learning may help in understanding the intricacies of relationships between inputs and the main outcome. They have the flexibility and the advantages of a built-in feature selection method, are able to handle many input variables without the need to minimize dimensionality, and can control overfitting by using out-of-bag validation.

This research combines five different approaches—Logistic Regression (LR), Random Forest (RF), Support Vector Machine (SVM), Naive Bayes (NB) and Artificial Neural Network (ANN) to examine the most suitable model for predicting the risk of T1D and explore factors affecting this disease in Saudi Arabian children.

All machine learning models have been utilised in a variety of studies to describe various systems such as environmental protection, agriculture as well as in the various areas of health [73–77], and hence they will be used and compared with the LR to identify the risk factors of T1D in children. The description of the individual ML models are summarised in the following sections. Additional references are provided for the detailed mathematical formula and theory behind the individual ML models. R statistical software was used to perform the analysis [78]. All code for data analysis associated with the current submission is available at https://github.com/Alazwari/R-code.git.

#### Logistic Regression (LR)

Logistic regression (LR) analysis is a commonly used statistical tool in medical research [79, 80]. It is the expansion of the linear regression model to use the probabilities of the two potential outcomes for classification problems. It is suitable for models involving disease state (diseased or healthy) and decision making (yes or no), and is thus widely applicable to health studies [81], including diabetes analysis of cases versus controls [82–84]. One of the important aspects of LR that makes it an effective and powerful tool in medical research, is the ability to determine the potential predictors [82, 85]. In addition, the advantages of LR are the simultaneous analysis of multiple explanatory variables, interactions, and reduction of the effect of confounding factors [82].

A LR model can be expressed in the form of:

$$\beta_{0}+\beta_{1}x_{1}+\beta_{2}x_{2}+\ldots +\beta_{n}x_{n}=log\left[P\left(Y\right)/1-P\left(Y\right)\right],$$

where *log*[*P*(*Y*)/1 − *P*(*Y*)] is the log (odds) of the outcomes, Y is the dependent variable, *x*_*i*_ are the independent variables, *β*_0_, *β*_1_, ‥*β*_*n*_ are the regression coefficients and *β*_0_ is the intercept.

Odds ratios (with 95% confidence intervals) will be used for assessing the KPIs of diabetes for significance. The LR assumptions have been checked and validated.

#### Random Forest (RF)

Decision trees are a commonly used machine learning tool because it is simple, easy to use, and interpretable for categorical data [86]. Several studies have been conducted to address the limitations of the traditional decision trees like their lack of robustness and suboptimal performance [87]. Based on these studies [86, 87], Random Forest (RF) was developed as an ensemble learning method in which the output of a number of weak learners, which could be a single decision tree, is improved by a voting scheme similar to other ensemble learning methods [88, 89]. RF can handle a large number of input variables without the need to reduce dimensionality because it has a built-in feature selection method [64, 75]. Also, in RF, out-of-bag validation can be used to control overfitting [75].

#### Support Vector Machine (SVM)

In the field of Machine Learning, Support Vector Machine (SVM), developed by Vladimir Vapnik, is among the most well-known and widely used algorithms [90]. SVM is a very effective method for building a classifier. Its aim is to create a decision boundary between two classes that allows for the prediction of labels using one or more feature vectors [91–94]. This decision boundary, called the hyperplane, is oriented to be as far away from the closest data points from each class as possible. These closest points are referred to as support vectors [88, 91, 92, 94]. In addition, the kernel approach, which allows us to model higher-dimensional, and non-linear models, is another advantage of SVM [92]. The kernel function chosen can have a significant impact on the performance of a SVM model. However, there is no way to know which kernel is optimum for a particular pattern recognition problem, this must be determined by trial [92].

In order to improve the SVM model accuracy, there are several parameters that need to be tuned. The main hyperparameter of the SVM is the kernel (linear, polynomial, radial basis function (RBF), and sigmoid kernel, Gaussian, Exponential, Hyperbolic tangent, ANOVA radial basis kernel, and Laplacian kernel). Every one of these kernels requires optimisation of one or more parameters such as cost, degree and gamma. The parameter cost controls over-fitting of the model by specifying tolerance for misclassification, gamma controls the degree of non-linearity of the model, and in the polynomial kernel, degree is the degree of the polynomial kernel function that controls the flexibility of the decision boundary. Higher degree polynomial kernels allow a more flexible decision boundary. In this study, we will use linear, polynomial, radial basis function (RBF), and sigmoid kernel. For each kernel, cross-validation is used to select the value of the parameters that optimise the SVM model.

#### Naive Bayes (NB)

Naive Bayes is a classification strategy based on Bayes Theorem and the idea that the existence of a specific feature in a class is unrelated to the presence of other features [95, 96]. It has been widely used for classification and prediction in many domains because it can be constructed quickly and easily from data. Also, it reduces space complexity, allowing quick inference, and often outperforms more complex learning algorithms in practice [97, 98]. The Naive Bayes Classifier provides a highly efficient probability estimation based on a simple structure, requiring only a small amount of training data to predict the classification parameters. It is based on two main assumptions: feature independence and the absence of hidden or latent properties [99].

#### Artificial Neural Network (ANN)

Artificial Neural Network (ANN) is a prediction approach used for finding a solution when other statistical methods are not suitable. The benefits of this method include the ability to learn from examples, fault tolerance (the property that allows an ANN to operate properly in the event one or more components are lost), and non-linear data forecasting, which all make it a commonly used statistical method [100]. The major benefit that ANN provides is the potential to distinguish hidden linear and nonlinear relationships, which often occur in high-dimensional and complex data sets [64, 101]. The ANN algorithms consist of input, hidden and output layer nodes where the nodes may also be referred to as “neurons” [102]. The number of input layer nodes represents the number of variables that describe the features evaluated, whereas the number of output layer neurons is equivalent to the number of levels of the outcome factor. The number of hidden layers and the number of neurons depend on the quantity of data and the complexity of the relationship. Every neuron in the hidden and output layer is linked by a corresponding numerical weight to all nodes in the proceeding layer [64, 103, 104]. It is these weights that determine the effect of neurons and impact the output.

### Models performance evaluation

In order to assess the models’ performance prediction, data are randomly divided into two subsets of 70% for training and 30% for the testing set to build and evaluate the LR, RF, SVM, NB, and ANN models. In addition, there are five common measures used in ML models [105, 106]: Accuracy, Sensitivity (Recall rate (R)), Receiver Operating Characteristic (ROC) which is equal to the Area Under the Curve (AUC), Precision rate (P), and F Score.

The diagnosis of T1D is a binary classification issue characterised by the ground truth that determines the performance of the prediction. The output of the prediction can be True Positive (TP), False Positive (FP), True Negative (TN), or False Negative (FN). The terms “positive case” and “negative case” were used to define the case group and the control group, respectively.

To obtain the ROC curve, the true positive rate (TPR) was plotted against the false positive rate (FPR). In this case, better performance is indicated by higher values of the (AUC) corresponding to the ROC curve.

The accuracy was also calculated and it is the proportion of the correctly predicted made for those with T1D and the controls divided by the total number of the dataset.

In addition to the correct identification of having T1D, we have considered the number of true positives (children who had T1D) using the Precision rate P and the number of diabetic children predicted using Recall rate R.

We also used the F Score to describe the efficiency of each model, with F Score close to 1 indicating better performance.

## Results

### Characteristics of the study sample

For this cohort, the range of birth year is from 2003 to 2020 and the median birth year was 2010. In addition, the demographic characteristics and potential risk factors for each of the case and control groups are shown in Tables 1 and 2. The results of the Chi-square (*χ*^2^) test to determine if there was a difference in the distribution of categorical variables between cases and controls are also shown in Tables 1 and 2.

**Table 1:** Characteristics of the study cohort for demographic, genetic, and nutritional history together with their corresponding frequency count and percentage for case and control groups. The hypothesis test results from a chi-square test of homogeneity in the distribution of categorical variables between the two groups together with their corresponding p-value are also presented. The significance level was set at *α* = 0.05, with statistically significant differences highlighted with an asterisk (*).

Variables	Cases (377)(33%)	Controls (765)(67%)	*χ* ^2^	P-value
**Demographic & Socioeconomic status**				
City: Al-Ahsa	109(28.91)	219(28.63)	0.07	0.965
Jeddah	159(42.18)	319(41.70)		
Riyadh	109(28.91)	227(29.67)		
Gender: Male	157(41.64)	324(42.35)	0.05	0.819
Female	220(58.36)	441(57.65)		
Residency: Rural	119(31.56)	95(12.42)	60.79	0.000*
Urban	258(68.43)	670(87.58)		
Income Status: High income: >12000 Saudi Riyals	8(212)	27(3.52)	9.57	0.042*
Upper-middle income:(>9000–12000) Saudi Riyals	19(5.03)	58(7.58)		
Middle income: (>6000–9000) Saudi Riyals	305(80.90)	621(81.17)		
Lower-middle income: (>3000–6000) Saudi Riyals	34(9.01)	47(6.14)		
Low income: ≤ 3000 Saudi Riyals	11(2.91)	12(1.56)		
**Genetic**				
Consanguineous parents: Yes	165(43.76)	350(45.75)	1.12	0.289
No	212(56.23)	415(54.24)		
First-degree of T1D:Father	13(3.44)	8(1.04)	49.92	0.000*
Mother	18(4.77)	16(2.09)		
Sibling	54(14.3)	36(4.70)		
None	292(77.45)	705(92.15)		
Second-degree of T1D: Yes	95(25.19)	165(21.56)	1.89	0.168
No	282(74.80)	600(78.43)		
**Nutritional history**				
Nutritional history: Breast feeding	64(16.97)	185(24.18)	18.48	0.000*
Introduction to cow’s Milk	82(21.75)	99(12.9)		
Both	231(61.27)	481(62.87)		
Introducing to solid food:(<6 months)	100(26.52)	221(28.88)	0.69	0.403
(>6 months)	277(73.47)	544(71.11)		

**Table 2:** Characteristics of the study cohort for obstetric history and maternal characteristics together with their corresponding frequency count and percentage for case and control groups. The hypothesis test results from a chi-square test of homogeneity in the distribution of categorical variables between the two groups together with their corresponding p-value are also presented. The significance level was set at *α* = 0.05, with statistically significant differences highlighted with an asterisk (*).

Variables	Cases (377)(33%)	Controls (765)(67%)	*χ* ^2^	P-value
**Obstetric history**				
Gestational length: very pre-term (<33)	5 (1.32)	28(3.52)	4.96	0.291
pre-term (33–36 weeks)	39(10.34)	76(9.67)		
early-term (37–38 weeks)	64(16.45)	128(16.73)		
full-term (39–40 weeks)	260(68.96)	499(66.01)		
post-term (>40)	13(3.44)	32(4.05)		
Birth delivery mode: Normal delivery	261(69.23)	573(74.90)	4.12	0.048*
Caesarean section (CS)	116(30.77)	192(25.09)		
Weight at birth’s: <2.5 Kg	115(30.50)	225(29.41)	14.69	0.000*
(2.5—3.5) Kg	219(58.09)	497(62.61)		
(3.5—4.0) kg	26(6.89)	31(4.05)		
>4.0 Kg	17(4.51)	12(1.56)		
Birth order: 1st	120(31.83)	216(28.23)	19.96	0.000*
2nd	70(18.56)	221(28.88)		
3rd	74(19.62)	165(21.56)		
Others (4th and more)	113(29.97)	163(21.30)		
**Maternal characteristics**				
Maternal Age at child’s birth: (<25 years)	80(21.22)	232(30.32)	15.45	0.000*
maternal age (25–35 years)	231(61.27)	447(58.43)		
maternal age (>35 years)	66(17.50)	86(11.24)		
Gestational diabetes: Yes	37(9.8)	43(5.6)	6.81	0.009*
No	340(90.18)	722(94.37)		
Pre-eclampsia: Yes	6(1.59)	23(3.00)	2.04	0.152
No:	371(98.40)	742(96.99)		
Maternal history of Asthma: Yes	14(3.71)	36(4.70)	0.59	0.440
No	363(96.28)	729(95.29)		

#### Demographic and socioeconomic status

In both case and control groups, a higher proportion of children were from Jeddah (42%), with approximately equal proportions from Al-Ahsa and Riyadh. More females (58%) were observed in both case and control groups than males (42%). Further, we can see that urban residents were significantly higher in the control (88%) than in case samples (68%) (*χ*^2^ = 60.8, p-value = <0.001). There were more consanguineous parents in the control group (46%) than in the case group (44%) but the difference was not statistically significant (p = 0.289).

#### Nutritional history

Breast feeding alone was reported by 24% of the control group and 17% of the case group. However, a similar proportion of children in the control and case groups were fed with both breastfeeding and cow’s milk (61% and 63%) (*χ*^2^ = 7.69, p-value = <0.001), and the timing of solid food introduction was also consistent across both groups. The majority of children in this study were self-reported as middle-income (81%).

#### Genetic (family history)

As expected from the prior literature, a higher percentage of children in the case group (22.55%) had a first-degree family history of T1D when compared to the control group (7.84%) (*χ*^2^ = 49.25, p-value = <0.001). The majority (75%) of both groups had no second-degree of family history of T1D.

#### Obstetric history

Full-term pregnancy (39–40 weeks) was the most common gestational age with (69%) and (66%) in the case and control groups respectively. There was a significant difference in the mode of birth delivery between the case and control group (*χ*^2^ = 4.12, p-value = 0.042). For the control group, 75% were normal birth and only 25% caesarean, whereas the caesarean rate increased to 31% in the case group with 69% by a normal birth. The child’s weight at birth showed similar percentages for all weight categories between case and control groups except for those with birth weight >4.0 kg. In the control group, only 2% had a weight >4.0 kg compared with the case group which had 5% with a birth weight >4.0 kg.

The frequency of being born 2nd was significantly lower in case group (19%) than control group (29%) and a higher proportion in the case group of a birth order of 4th or higher (*χ*^2^ = 19.96, p-value = <0.001).

#### Maternal characteristics

The maternal age group of 25–35 was the most common in both cases and control groups with (61%) and (58%) respectively. Mothers aged over 35 years were reported more in case samples (18%) than in control samples (11%) (*χ*^2^ = 15.45, p-value = <0.001). A higher proportion of cases (9.8%) reported mothers that experienced gestational diabetes (*χ*^2^ = 6.81, P-value = 0.009). For pre-eclampsia and maternal history of asthma, there were no significant differences between case and control groups.

### Models performance

**Fig 1:**
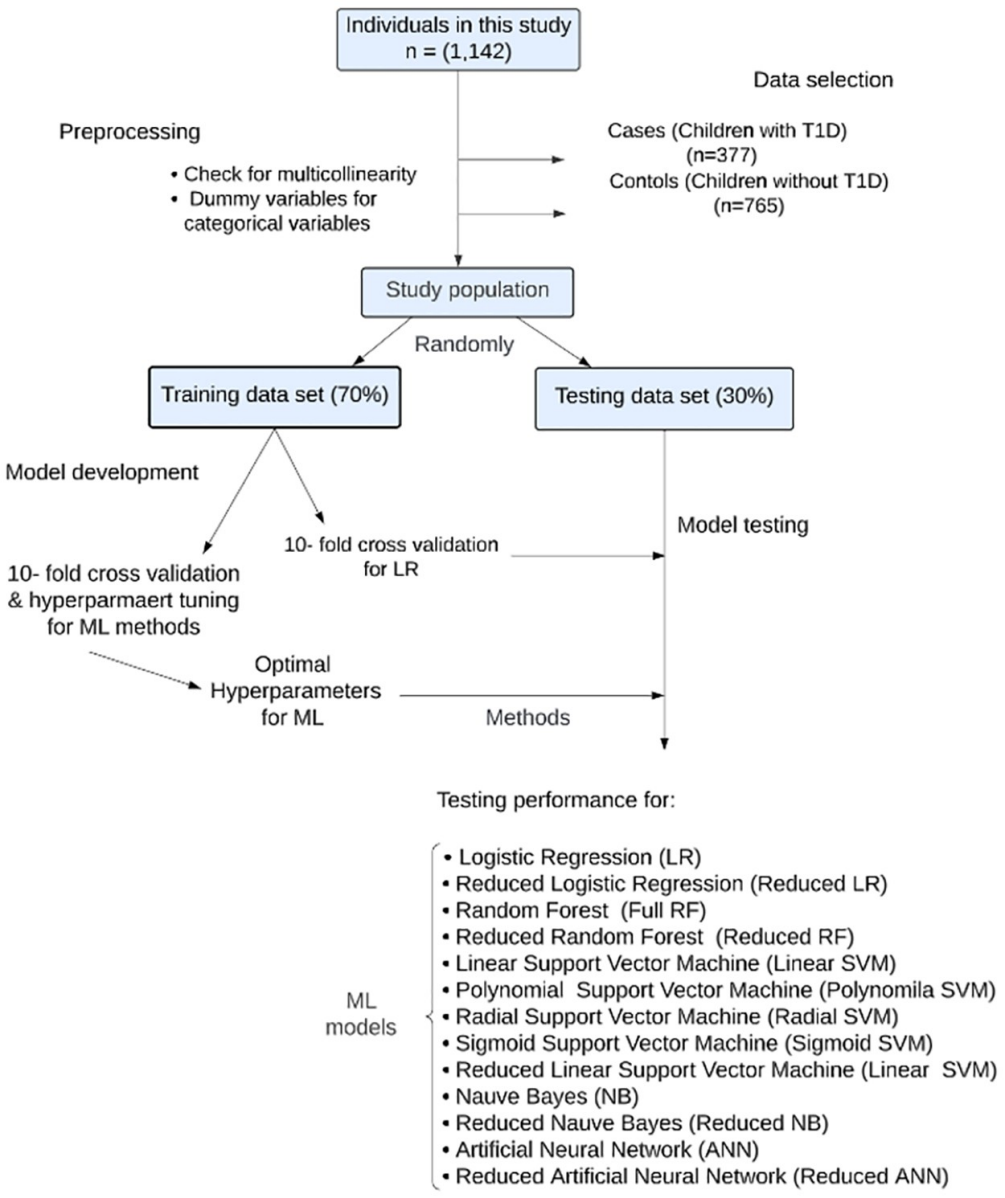
Flowchart of models performance.

Data was randomly divided into two subsets of 70% for training and 30% for the testing set to build and evaluate the LR, RF, SVM, NB, and ANN models. Also, we adopted a k-fold cross-validation approach to assess the models’ performance where k denotes the number of groups in which data is split and k = 10. In addition, for ML models, hyperparameters were tuned in order to optimize the ML models (Fig 1). Table 3 shows a summary of the result of the models’ performance on the testing set and is described below.

**Table 3:** 10 fold Cross-validation results of LR, RF, SVM, NB, and ANN.

Model	Accuracy	CI	Sensitivity(Recall)	Specificity	Precision	F Score	AUC
Full LR	0.77	(0.7116, 0.8264)	0.70	0.82	0.70	0.70	0.83
Reduced LR	0.75	(0.6825, 0.8016)	0.59	0.82	0.61	0.60	0.78
Full RF	0.75	(0.7019, 0.8181)	0.61	0.83	0.64	0.62	0.81
Reduced RF	0.73	(0.6681, 0.7891)	0.55	0.83	0.59	0.56	0.77
SVM(Linear)	0.75	(0.6825, 0.8016)	0.65	0.80	0.60	0.62	0.80
SVM(Polynomial)	0.75	(0.6874, 0.8058)	0.59	0.81	0.60	0.60	0.77
SVM(Radial)	0.73	(0.6729, 0.7933)	0.58	0.81	0.60	0.59	0.79
SVM(Sigmoid)	0.75	(0.6922, 0.8099)	0.65	0.81	0.61	0.63	0.79
Reduced SVM (Linear)	0.72	(0.6729, 0.7933)	0.50	0.85	0.59	0.52	0.72
NB	0.75	(0.6777, 0.7975)	0.63	0.81	0.60	0.61	0.75
Reduced NB	0.72	(0.6586, 0.7808)	0.50	0.83	0.58	0.53	0.73
ANN:one hidden layer (14 nodes)	0.70	(0.6395, 0.7640)	0.52	0.80	0.60	0.56	0.70
ANN:two hidden layers (12 nodes)	0.70	(0.6282, 0.7448)	0.58	0.77	0.60	0.57	0.72
Reduced ANN:two hidden layers	0.65	(0.5877, 0.7172)	0.54	0.70	0.50	0.51	0.66

Table 3 shows the detailed performance results of the LR and ML classification methods. Logistic regression (LR) based on all variables (full model) achieved a prediction Accuracy (0.77, 95% CI [0.7116, 0.8264]), Sensitivity (Recall), Precision, and F Score of (0.70), and AUC (0.83). The interactions between the independent variables were explored to further improve the LR model. However, due to the small number of observations in some levels of the independent variables, such as the maternal history of asthma (n = 14) (Table 2), the results did not show further improvement for this dataset. In addition, we built the reduced LR based on the most significant factors (based on their corresponding P-value) presented in (Table 4) and (S1 Table). The reduced LR achieved lower values of Accuracy (0.75, 95% CI [0.6825, 0.8016]), Sensitivity (Recall) (0.59), Precision (0.61), F Score (0.60), and AUC (0.78) compared with the full LR model.

**Table 4:** Risk factors of T1D in children based on the logistic regression model.

Variables	OR	CI	P-value
Intercept	0.014	(0.003, 0.058)	0.000*
City (Jeddah)	1.618	(1.111, 2.370)	0.012*
Residency (Rural)	3.745	(2.604, 5.415)	0.000*
Nutrition history (Introduction to cow’s milk)	2.928	(1.847, 4.678)	0.000*
Nutrition history (Both)	1.580	(1.095, 2.301)	0.016*
First degree of T1D (Father)	6.181	(2.355, 17.189)	0.000*
First degree of T1D (Siblings)	3.708	(2.282, 6.083)	0.000*
First degree of T1D (Mother)	2.639	(1.228, 5.707)	0.012*
Second degree of T1D (Yes)	1.598	(1.146, 2.225)	0.005*
Birth weight (>4 kg)	3.114	(1.367, 7.356)	0.007*
Maternal age at child’s birth (25–35 years)	1.691	(1.169, 2.463)	0.006*
Maternal age at child’s birth (>35 years)	2.420	(1.393, 4.217)	0.002*
Income status (Low income)	3.063	(0.828, 11.949)	0.098
Delivery birth mode (CS)	1.369	(0.997, 1.877)	0.051
Birth order (1st)	1.520	(0.987, 2.350)	0.057
Birth year	0.992	(0.952, 1.035)	0.739
City (Al-Ahsa)	1.323	(0.876, 2.002)	0.183
Gender (Female)	1.147	(0.863, 1.527)	0.346
Consanguineous parents (Yes)	0.964	(0.727, 1.276)	0.798
Solid food (less than 6 months)	0.810	(0.586, 1.114)	0.198
Income status (Lower-middle income)	2.275	(0.854, 6.566)	0.111
Income status (middle income)	1.778	(0.768, 4.583)	0.201
Income status (Upper-middle income)	1.170	(0.421, 3.464)	0.768
pregnancy length (full-term [39–40 weeks])	1.385	(0.672, 2.998)	0.390
pregnancy length (early-term [37–38 weeks])	1.156	(0.519, 2.681)	0.727
pregnancy length (pre-term [36–33 weeks])	1.219	(0.513, 2.992)	0.658
pregnancy length (very pre-term [<33 weeks])	0.427	(0.105, 1.550)	0.209
Gestational diabetes (Yes)	1.370	(0.801, 2.329)	0.245
Birth order (2nd)	0.737	(0.476, 1.140)	0.171
Birth order (3rd)	0.903	(0.590, 1.381)	0.640
Pre-eclampsia (Yes)	0.527	(0.178, 1.357)	0.209
Asthma (Yes)	0.824	(0.394, 1.636)	0.593
Birth weight (<2.5 kg)	1.318	(0.946, 1.834)	0.101
Birth weight (3.5–4.0 kg)	1.979	(1.059, 3.667)	0.347

*Significant at p-value <0.05

**Fig 2:**
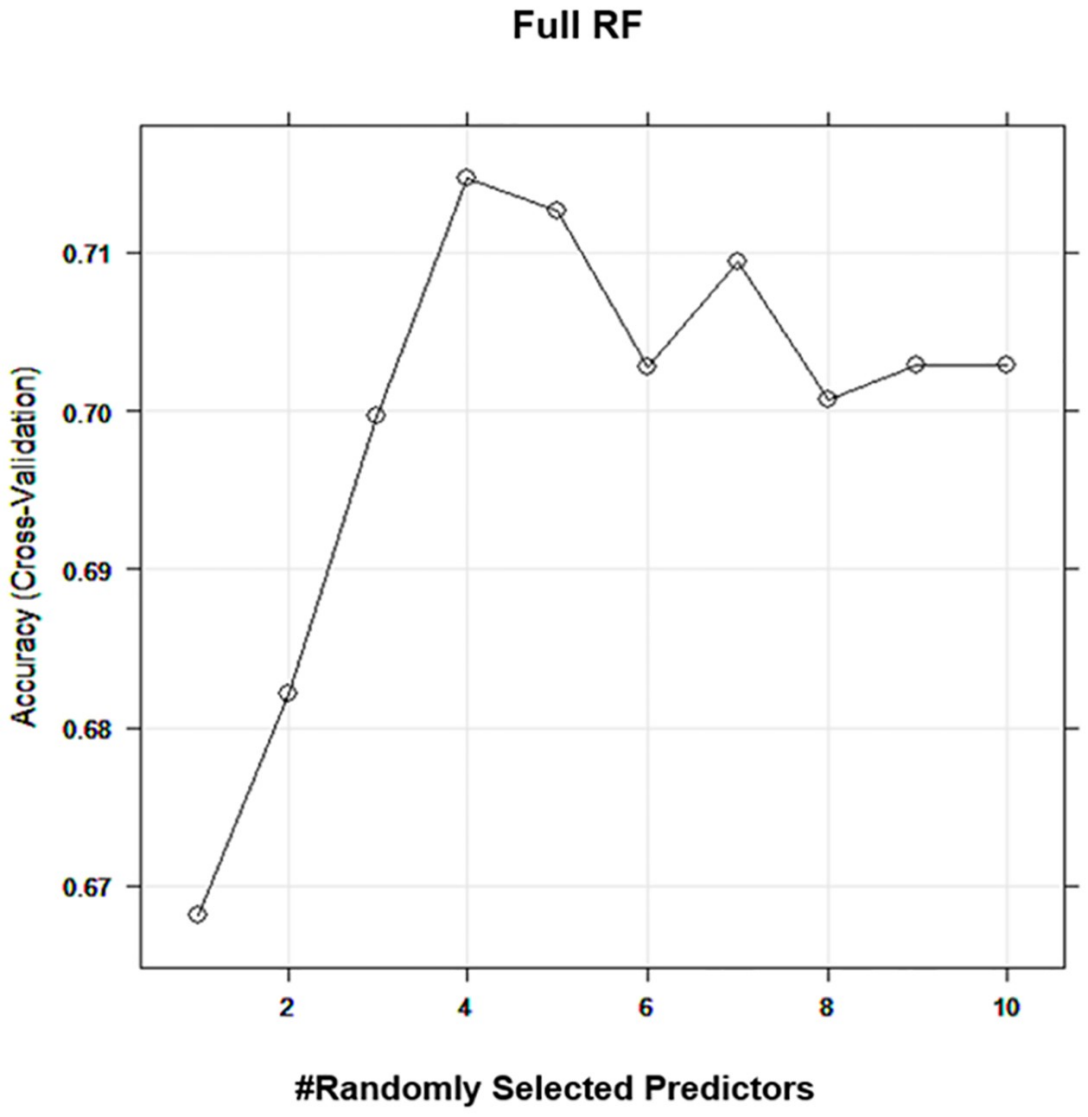
Tuning hyperparameter for full RF model. The best mtry for the full RF model based on the 10-fold cross-validation.

Random Forest (RF) models have been built using all independent variables. In addition, the hyperparameter that controls the split variable randomization feature of RF is often referred to mtry. This is the number of variables randomly sampled as candidates at each split, and it helps to balance the trade-off between a low correlation reasonable strength. Through 10-fold cross-validation based on the training data, we observed that the best mtry for the full RF is 4 (Fig 2).

Table 3 shows the Full RF achieved Accuracy (0.75), Sensitivity (Recall) (0.61), Precision (0.64), F Score (0.62), and AUC (0.81).

**Fig 3:**
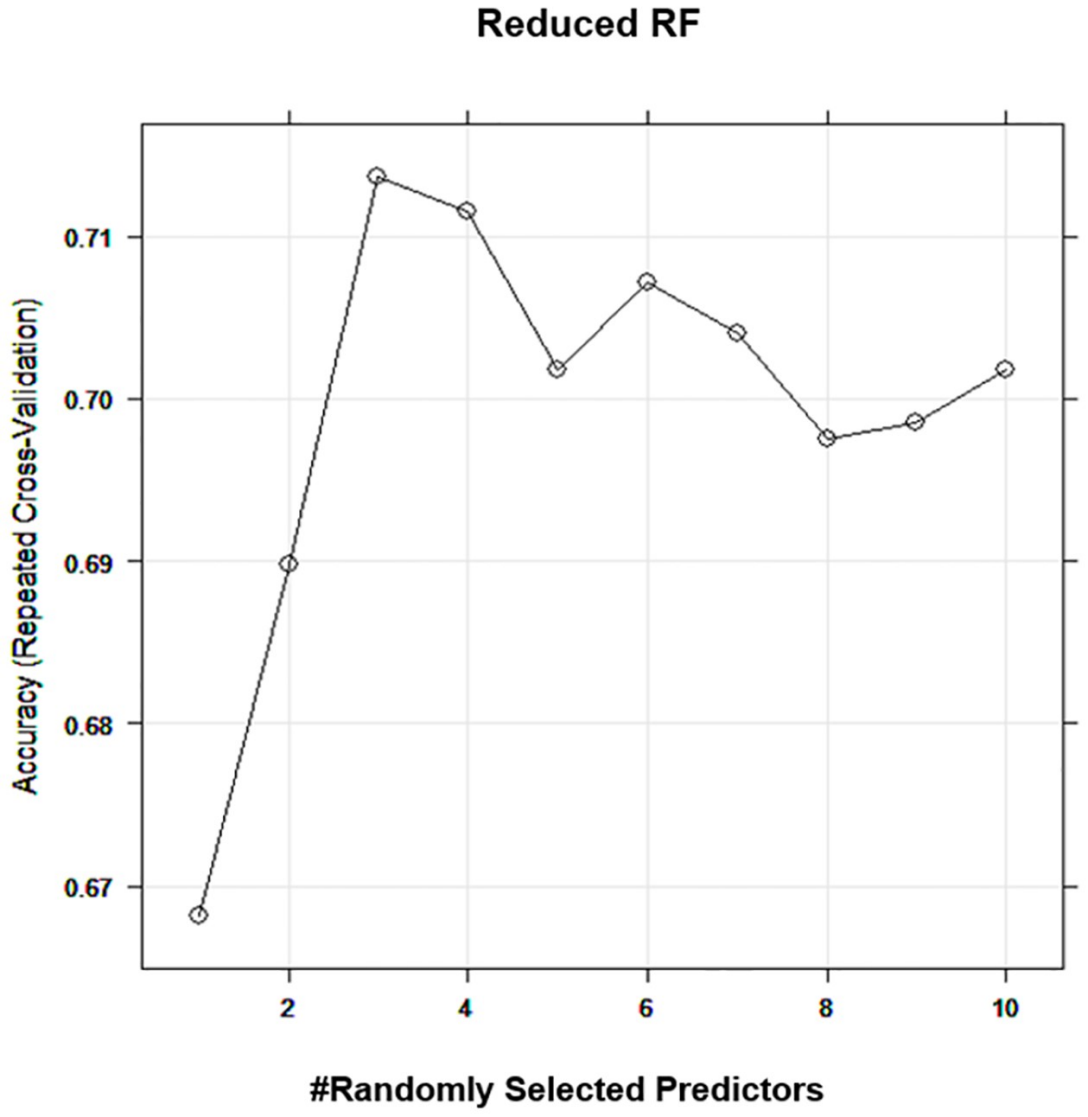
Tuning hyperparameter for reduced RF model. The best mtry for the reduced RF model based on the 10-fold cross-validation.

Significant variables from the full models (S1 Table) and (S1 Fig) are used to build the reduced RF. Similar to the full model, the hyperparameter was considered in reduced RF and we found that the best mtry for the reduced RF is 3 (Fig 3).

The results in Table 3 show that the reduced RF achieved Accuracy (0.73), Sensitivity (Recall) (0.58), Precision (0.60), F Score (0.56), and AUC (0.80).

In Support Vector Machine (SVM), the main hyperparameter is the kernel, the mathematical function used to transform data that cannot initially be separated linearly, was considered to improve the SVM model accuracy. SVM can use different kernels and each of these kernels requires one or more parameters, including cost, degree, and gamma. Four different SVM kernels were explored linear, polynomial, radial, and sigmoid. The optimal model was found with the linear SVM with all variables, and it was based on a cost parameter of 10. However, the best polynomial SVM was based on the degree of 3 and the cost of 1. For the radial SVM, the cost of 2 and gamma of 0.1 was found to be optimal and the sigmoid SVM used a gamma of 0.1, and a cost of 1. The results of the evaluation of SVM models (Table 3) indicated that the linear and sigmoid kernel functions achieved a high AUC of (0.80 and 0.79), Sensitivity of 0.65, and F Score of (0.62 and 0.63) compared with other kernel functions. To further improve the best SVM model (linear SVM), the reduced model has been built based on the significant variables (S1 Table) and (S2 Fig). However, the reduced linear SVM model has the lowest values of Sensitivity and F Score (0.50 and 0.52) respectively compared with the full SVM models.

In addition, we tuned the hyperparameter in Naive Bayes (NB) using the kernel density estimation. As a result, the NB model achieved an Accuracy (0.75), Sensitivity (0.63), F Score (0.61), and AUC (0.75). The reduced NB model also was considered and built using the significant variables identified (S1 Table) and (S3 Fig) for further improvement. The reduced NB model achieved Accuracy (0.72), Sensitivity (0.50), F Score (0.53), and AUC (0.73) (Table 3). Therefore, the full NB outperforms the reduced NB model.

**Fig 4:**
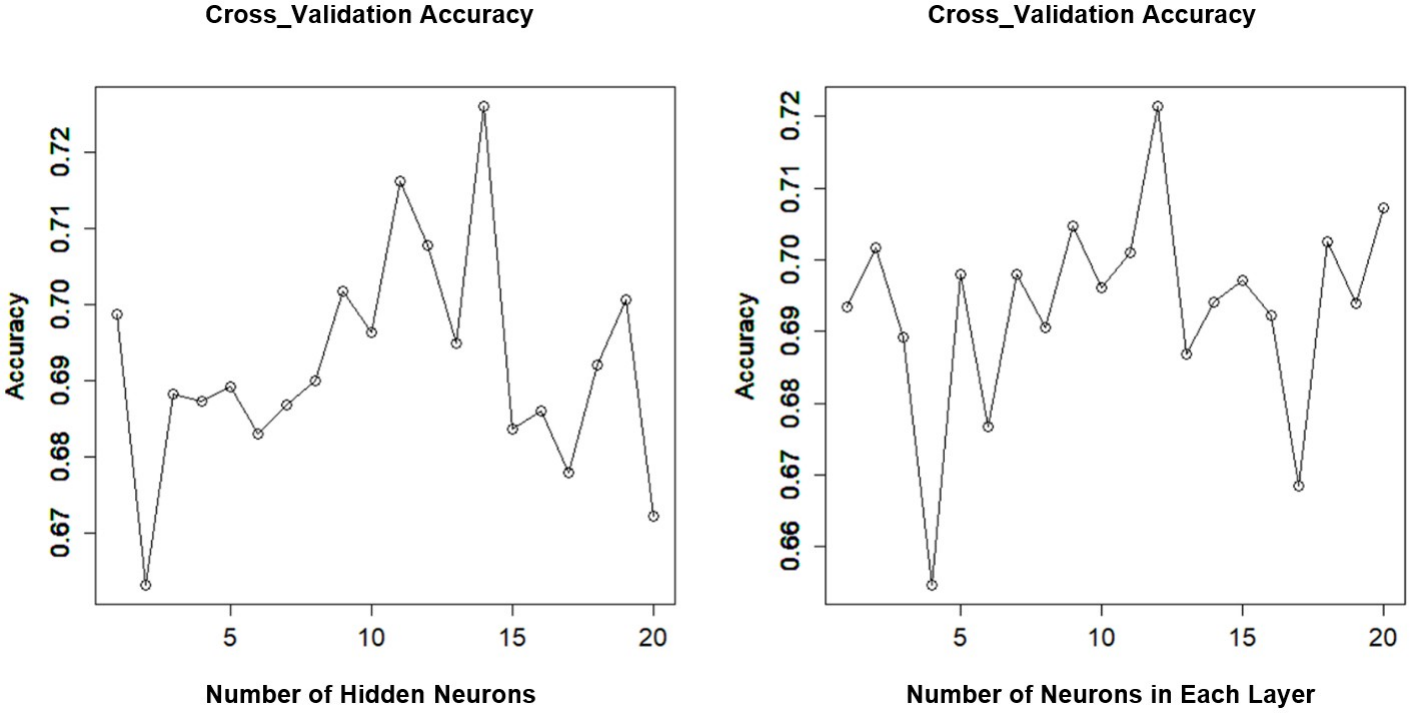
Tuning hyperparameter for ANN models. ANN with one hidden layer (a) and ANN with two hidden layers (b).

For ANN the number of neurons in the hidden layers is one of the hyperparameters to tune. There is no standardised approach for determining the number of neurons in a hidden layer [107]. The number of hidden layer neurons varies from problem to problem, and it depends on the number and quality of training patterns [64, 108]. We used 10-fold cross-validation to determine the optimal number of neurons in hidden layers. A similar accuracy and AUC were observed between ANN models with one and two hidden layers. (Fig 4) shows that the highest accuracy is at 14 neurons when using one hidden layer and at 12 neurons when using two hidden layers.

**Fig 5:**
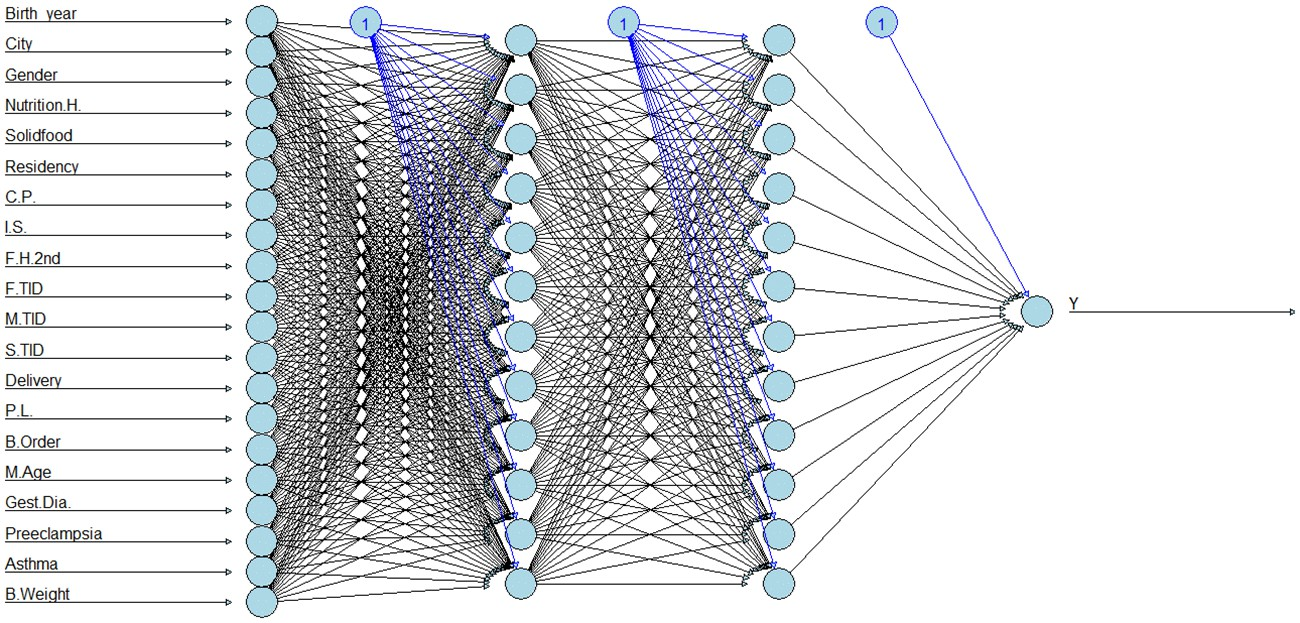
An optimal ANN model with 2 hidden layers with all input variables. P.L: pregnancy length, F.H.2nd: Family history of T1D with a second-degree relative, I.S.:Income Status, C.P.: Consanguineous Parents, M.Age: Maternal Age, B.Weight: Birth weight, B.Order: Birth Order.

Both ANN models with one and two hidden layers have the same Accuracy (0.70) and Precision (0.60) and similar F Score (0.56 and 0.57), and AUC (0.70 and 0.72) respectively. However, the ANN with two hidden layers achieved a high value of Sensitivity (0.58) compared with ANN with one hidden layer (0.52). So, the optimal ANN model was with two hidden layers and 12 neurons in each layer (Fig 5).

Furthermore, the reduced ANN model with two hidden layers was also built using the significant variables from (S1 Table) and (S4 Fig). The reduced ANN model achieved lower performance with Accuracy (0.65), Precision (0.50), F score (0.51), and AUC (0.66) (Table 3).

### Models comparison

**Fig 6:**
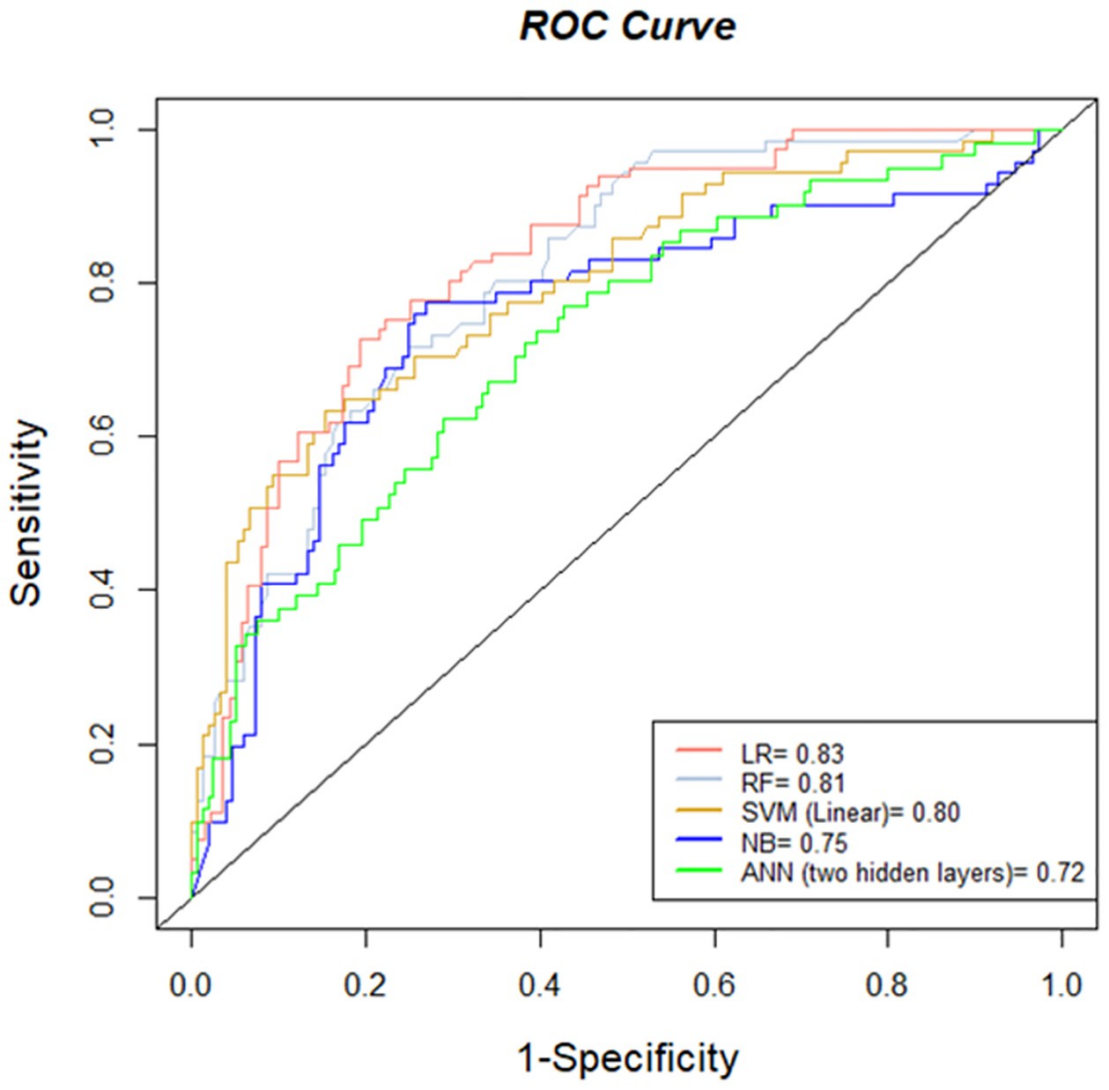
ROC curve and AUC for the best-performing model of different approaches.

As shown in Table 3, the full LR model has the highest value of Sensitivity, Precision, and F Score (0.70) compared to all other models. Whereas the reduced SVM, reduced NB, and ANN with one hidden layer and reduced ANN models showed weaker model performance with Sensitivity (0.50, 0.50, 0.52 and 0.54) and AUC (0.72,73, 0.70, and 0.66) respectively. The best performing models from every approach are shown in (Fig 6). LR has the highest value of AUC (0.83) followed by full RF which achieved AUC of 0.81. SVM linear showing a slightly lower AUC of 0.80. Therefore, LR (logistic regression) yields a better Accuracy, Sensitivity, Precision, F Score, and AUC while showing similar performance for Specificity as the machine learning models to predict childhood T1D in Saudi Arabia. An additional figure of the ROC curve performance for all other models is located in the Supporting information section (S5 Fig).

### Selection of the significant variables

Logistic regression was subsequently used to estimate the importance of each predictor. The variables that were identified to be significantly associated with T1D included: rural residency, introduction to cow’s milk, first-degree T1D of father, mother, and siblings, presence of second-degree T1D, birth weight more than 4 kg, and maternal age of 25–35 and over 35 years at child’s birth (Table 4). In addition, the risk factors such as income status (low income), birth delivery(CS), and birth order(first) (Table 4), did not reach the level of statistical significance (P-value <0.05) but they show a trend towards a relationship.

In addition, the significant variables identified by LR were also identified as significant variables by one or more of the ML models (S1 Table). For example: residency, and maternal age at child birth were identified as significant by all models. The first degree of T1D (siblings) was identified as a significant variable by LR and three ML models (SVM, NB, and ANN).

S1–S4 Figs in (Supporting information section) show the variables importance based on each ML model.

## Discussion

This study has several strengths. To the best of our knowledge, it was the largest study to investigate the association between the environmental and family history factors of T1D in children in Saudi Arabia. We have used local data to create a more robust and clinically relevant predictive model of T1D.

Further different statistical and ML approaches were used to find the optimal model to predict T1D in children and identify its significant KPIs.

To ensure a broad representation of the diverse population was covered, de-identified data of 1,142 children collected from three cities in Saudi Arabia were used for this case-control study. Having access to a statically sufficient sample size (1,142), we have compared the performance of LR and modern ML approaches to predict T1D. The efficacy of models was assessed using multiple criteria (Accuracy, Sensitivity, Precision, F Score, and AUC). The results presented in Table 3 showed that LR has the best performance with the highest values of Accuracy (0.77), the same value of Sensitivity, Precision, and F score (0.70), and AUC (0.83). This was followed by the performance of full RF and full SVM (linear) with AUC (0.81 and 0.80) and Accuracy (0.75 and 0.75) respectively.

This was consistent with the previous study [109] where the authors concluded that LR “was as good as” ML in predicting major chronic diseases. The comparison results outlined above were obtained after tuning hyperparameters for each ML model and 10-fold cross-validation for LR and ML models. Subsequently, LR as the best performing model was used to identify the significant KPIs of childhood T1D.

Our study has investigated both environmental and family history as risk factors for T1D. The results presented in Table 4 show that urban residence is linked to lower incident rates compared to rural areas. This result supports previous findings outlined in [17, 18] but it is in contrast with the findings of [15, 16, 110]. This contrast could be due to the differences in study design and the country of origin under investigation. For example, in [15] only the cases group was considered, and in [16] the population densities were categorised into four levels.

Our result has identified early exposure to cow’s milk as one of the significant KPIs (OR = 2.92, 95% CI [1.84, 4.67], P = 0.000). This is in agreement with the finding in the previous studies [25, 26]. However, other studies [111, 112] have reported that early exposure to cow’s milk had no association with T1D (OR = 1.06, 95% CI [0.36, 3.09], p = 1.000) and (OR = 0.85, 95% CI [0.61, 1.18], p = 0.332) respectively.

Our study shows that the risk of T1D is significantly associated with a positive family history of T1D in agreement with the previous study [14, 113]. The results presented in Table 4 indicated that T1D in first and second-degree relatives increases the risk of T1D in children. Specifically, the chance of developing T1D for children whose father is affected by T1D is 6.18 times higher (OR = 6.18, 95% CI [2.35, 17.18]). Also, the risk of T1D for children whose siblings are affected by T1D is 3.70 higher (OR = 3.70, 95% CI [2.28, 6.08]).

Moreover, our results indicated that child’s birth weight >4 kg was a risk factor for childhood T1D whereas a low birth weight had no statistically significant impact. This is in agreement with the previous studies [35, 38, 114]. However, the authors in [36] showed that there is no association between a child’s high birth weight and T1D (OR: 1.01, 95%CI: 0.96–1.05), and in [115] the authors reported a significantly lower risk of T1D in children with low birth weight (<2.5 kg) (OR: 0.82, 95%CI: 0.67–0.99). These results could be due to the difference in the definition of the birth weight reference levels. The reference levels of birth weight in [36] were (3.0—4.0 kg) and in [115] (3.0- 3.5 kg) which are different to (2.5—3.5 kg) considered in our study. The reference level (2.5—3.5 kg) considered in our study is the recommended normal weight for the children T1D [38, 71, 72].

It is worth mentioning that merging the categories of (3.5—4kg) and (>4 kg) provided a more reasonable sample size, consequently, a narrower confidence interval (95% CI [1.07, 3.24]) but the value of (OR = 1.86) confirms that birth weight is a significant risk factor. The child’s birth weight >4 kg is identified as high risk, therefore it was decided to include this category in the analysis (Table 4) to be consistent with the literature and recommendation of the American College of Obstetricians and Gynaecologists definitions. [38, 72]. Other obstetric factors such as gestational length (gestational age) were not associated with childhood T1D in our study in line with prior findings from study conducted in Israel [116] and conflicts with previous studies in [35–37, 114, 115] that demonstrated the preterm birth (34–36 weeks) has a negative impact on T1D.

Our results showed that maternal T1D diabetes, and maternal age at child’s birth (greater than 25 years) were significant factors of childhood T1D. This is consistent with the results presented in [35, 37]. However, other maternal characteristics such as gestational diabetes, pre-eclampsia, and maternal history of asthma were not identified as significant factors of childhood T1D in our study but were shown as significant factors in [23, 37, 41, 42, 117]. This may reflect the small number of observations related to these characteristics in our study. Other risk factors such as the weight of the mother at childbirth [118] were not included in the medical records of Saudi Arabia children, which is a limitation of using secondary data in this study.

This should be considered as a key factor in future data collection for research, particularly as female obesity has increased in Saudi Arabia over the last decade [119]. A further limitation of the current study is the use of controls from existing hospital records. Although all controls were identified as free from T1D, they had attended a clinic for medical treatment and hence may not fully represent the health population with no medical clinic attendance.

The results presented here show the importance of collecting and monitoring the significant KPIs identified in this study to improve public health outcomes. The creation of a unified electronic health record linking all hospitals in the country would increase the efficacy of data collection (sample size, diversity and monitoring of pregnancy variables, birth characteristics, and child development over time) and enable further refinement of our predictive T1D model.

## Conclusion

This study is the largest case-control study to investigate the association between environmental and family history factors of childhood T1D in Saudi Arabia. The country has an increasing incidence rate of T1D in children, currently the 5th highest rate in the World. With regards to the total number of children with T1D, Saudi Arabia ranks 7th in the World. Despite this remarkably high incidence, there is a lack of targeted, comprehensive T1D research considering the diversity of the Saudi Arabian population compared to developed countries.

Existing T1D research in Saudi Arabia is limited to small sample sizes, a single center, a single city or region of the country, or a small number of associated factors. In this study, secondary data from a total of (1,142) individual medical records collected from three cities located in different regions of Saudi Arabia have been used in the analysis to represent the country’s diverse population. Both cases and matched controls data (birth year, gender, and location) are used to create a more robust and clinically relevant model by controlling confounders. Also, a wide range of potential KPIs suggested in the literature have been included in this study.

We have utilised several approaches to find the optimal model, including modern Machine learning methods, as their application has increasingly gained interest in the healthcare domain. Specifically, we used logistic regression, Random Forest, Support Vector Machine, Naive Bayes, and Artificial Neural Network with local data to find the most clinically relevant optimal model to predict T1D in children. The optimal model was then used to identify the significant KPIs of T1D in children aged (0–14 years).

The analysis of environmental and family history factors revealed significant differences across demographic and socioeconomic status, genetic and disease history, nutrition history, obstetric history, and maternal characteristics. The results show that most children (cases = 68% and controls = 88%) are from urban areas of Saudi Arabia, 23% (cases) and 8% (control) have a family history of T1D (first-degree relative), and 31% of the cases group were delivered through a caesarean section which was higher than the control group (*χ*^2^ = 4.12, P = 0.042). Also, the percentage of mothers older than 35 years was higher in case samples (18%) than in control samples (11%) (*χ*^2^ = 8.59, P = 0.003).

Comparing the performance of different models based on Accuracy, Sensitivity, Precision, F Score, and AUC, it is shown that the logistic regression model outperforms the other models with Accuracy = 0.77, Sensitivity, F Score and Precision of 0.70, and AUC = 0.83. This was followed by the performance of the full Random Forest and the full Support Vector Machine (linear) models with AUC (0.81 and 0.80) and Accuracy (0.75 and 0.75) respectively.

The comparison results outlined above were obtained after tuning hyperparameters for each ML model and 10-fold cross-validation for all LR and ML models. To investigate whether we could further enhance the prediction Accuracy and simplify the models, significant variables identified by the individual full model were used to build the reduced models. The performance of the reduced model in terms of efficacy criteria was less impressive compared with the full models.

The full logistic regression was then employed to identify the most significant KPIs of childhood T1D in Saudi Arabia. The results show that the most significant KPIs are early exposure to cow’s milk (OR = 2.92, P = 0.000), birth weight >4 Kg (OR = 3.11, P = 0.007), residency(rural) (OR = 3.74, p = 0.000), family history of T1D in the first degree (father (OR = 6.18, P = 0.000), and siblings (OR = 3.07, P = 0.000)), and second degree (OR = 1.59, P = 0.005), and maternal age (25–35 years) and greater than 35 years with (OR = 1.69, P = 0.006) and (OR = 2.42, P = 0.002) respectively.

This study makes a significant contribution to Saudi Arabian childhood T1D research by providing the most clinically relevant optimal model to predict T1D in children and identifying its associated KPIs using local data. The results presented in this paper will also assist healthcare providers in collecting and monitoring the influential KPIs data. This would enable the initiation of suitable intervention strategies to reduce the disease burden and potentially slow the incidence rate of childhood T1D in Saudi Arabia.

Furthermore, the research demonstrates that access to a nationwide electronic health record database linking all hospitals in the country could further improve the efficacy of the predictive model with respect to the population diversity attributes. The results presented in this paper have also contributed to filling the gap in childhood T1D research of non-European populations.

## Supporting information

S1 Table Significant variables based on each full model. (DOCX)

S1 Fig Variables importance based on RF model. P.L: pregnancy length, F.H.2nd: Family history of T1D with a second-degree relative, I.S.:Income Status, C.P.: Consanguineous Parents, M.Age: Maternal Age, B.Weight: Birth weight, B.Order: Birth Order.
(TIF)

S2 Fig Variables importance based on SVM model. P.L: pregnancy length, F.H.2nd: Family history of T1D with a second-degree relative, I.S.:Income Status, C.P.: Consanguineous Parents, M.Age: Maternal Age, B.Weight: Birth weight, B.Order: Birth Order.
(TIF)

S3 Fig Variables importance based on NB model. P.L: pregnancy length, F.H.2nd: Family history of T1D with a second-degree relative, I.S.:Income Status, C.P.: Consanguineous Parents, M.Age: Maternal Age, B.Weight: Birth weight, B.Order: Birth Order.
(TIF)

S4 Fig Relative importance of each variable using Olden’s algorithm. P.L: pregnancy length, F.H.2nd: Family history of T1D with a second-degree relative, I.S.:Income Status, C.P.: Consanguineous Parents, M.Age: Maternal Age, B.Weight: Birth weight, B.Order: Birth Order.
(TIF)

S5 Fig ROC curve and AUC for all other models. (TIF)
