# Supplementary material for: Predicting the development of T1D and identifying its Key Performance Indicators in children; a case-control study in Saudi Arabia
Source: PLoS One. 2023 Mar 1;18(3):e0282426. doi: 10.1371/journal.pone.0282426 (PMC9977054; doi:10.1371/journal.pone.0282426)
Supplement: S1 Table — (DOCX) [file pone.0282426.s001.docx]

**S1 Table. Significant Variables based on each full model**

| Variables | LR | RF | SVM | NB | ANN |
| --- | --- | --- | --- | --- | --- |
| Residency | * | * | * | * | * |
| Maternal age at child’s birth | * | * | * | * | * |
| Birth weight | * | * |  |  | * |
| First degree of T1D (Siblings) | * |  | * | * | * |
| Second degree of T1D | * |  | * | * | * |
| Birth order | * | * | * | * | * |
| City | * | * |  |  | * |
| Nutrition history | * | * |  |  | * |
| Delivery birth mode | * |  | * | * | * |
| Income status | * | * | * | * |  |
| First degree of T1D (Father) | * |  |  |  | * |
| First degree of T1D (Mother) | * |  |  |  | * |
| Gestational diabetes |  |  | * | * |  |
| Pregnancy length |  | * |  |  |  |
| Consanguineous parents |  | * |  |  | * |
| Solid food |  | * |  |  |  |
