# Supplementary figures and images for: Predicting the development of T1D and identifying its Key Performance Indicators in children; a case-control study in Saudi Arabia
Source: PLoS One. 2023 Mar 1;18(3):e0282426. doi: 10.1371/journal.pone.0282426 (PMC9977054; doi:10.1371/journal.pone.0282426)

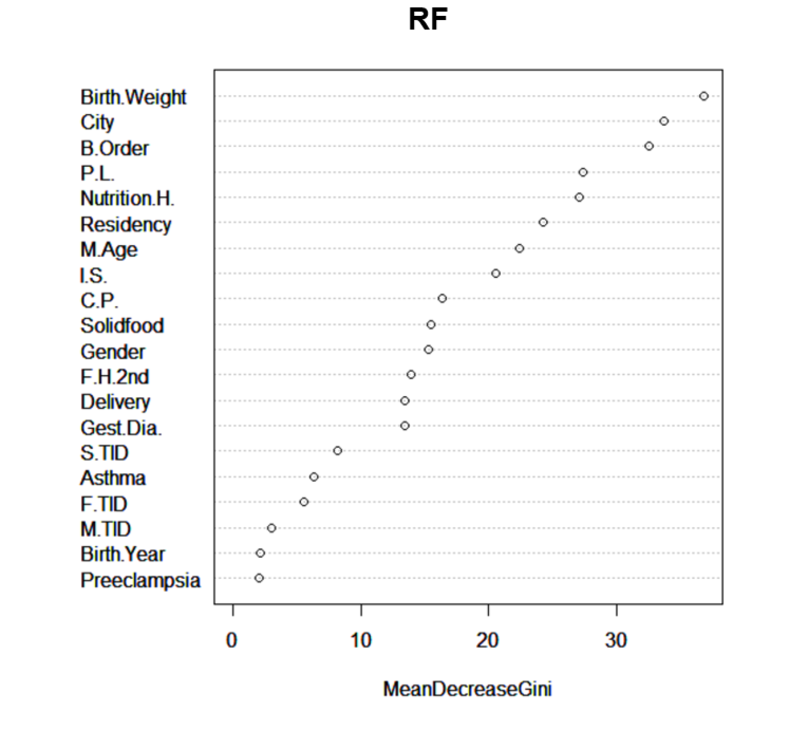

Supplement: S1 Fig — P.L: pregnancy length, F.H.2nd: Family history of T1D with a second-degree relative, I.S.:Income Status, C.P.: Consanguineous Parents, M.Age: Maternal Age, B.Weight: Birth weight, B.Order: Birth Order. (TIF) [file pone.0282426.s002.tif]

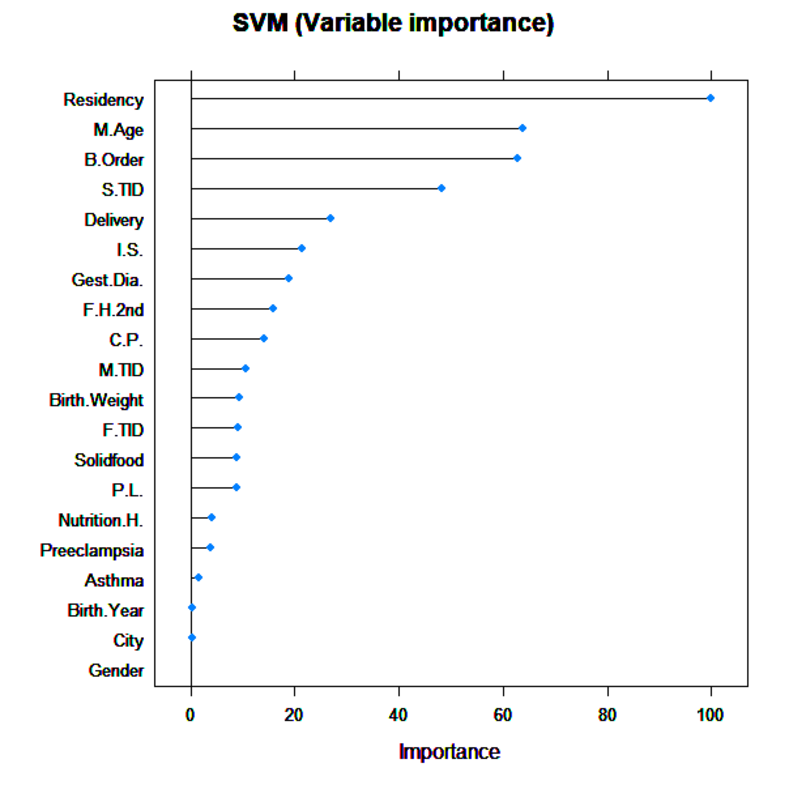

Supplement: S2 Fig — P.L: pregnancy length, F.H.2nd: Family history of T1D with a second-degree relative, I.S.:Income Status, C.P.: Consanguineous Parents, M.Age: Maternal Age, B.Weight: Birth weight, B.Order: Birth Order. (TIF) [file pone.0282426.s003.tif]

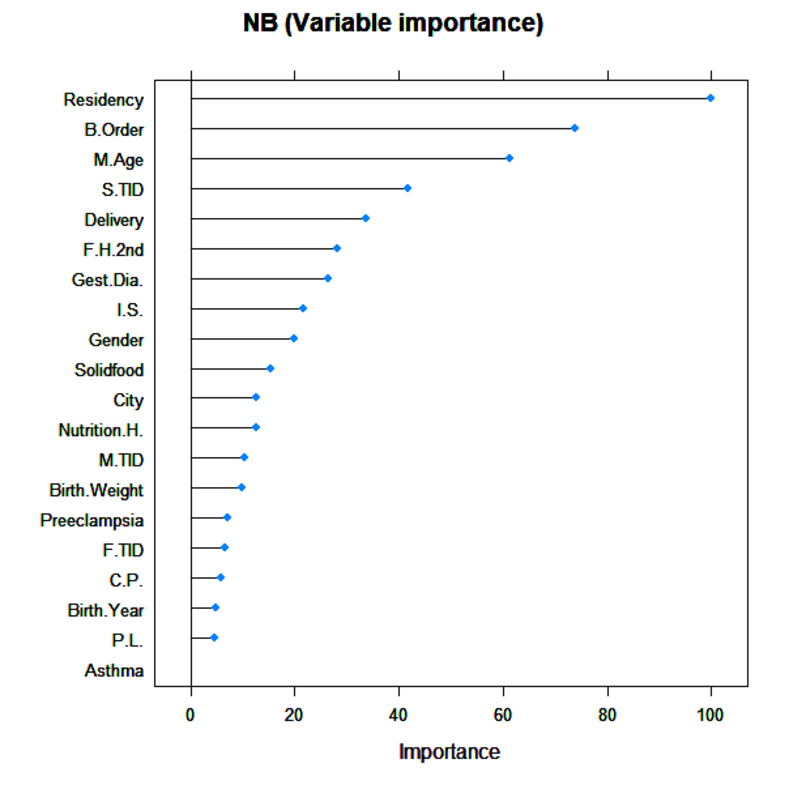

Supplement: S3 Fig — P.L: pregnancy length, F.H.2nd: Family history of T1D with a second-degree relative, I.S.:Income Status, C.P.: Consanguineous Parents, M.Age: Maternal Age, B.Weight: Birth weight, B.Order: Birth Order. (TIF) [file pone.0282426.s004.tif]

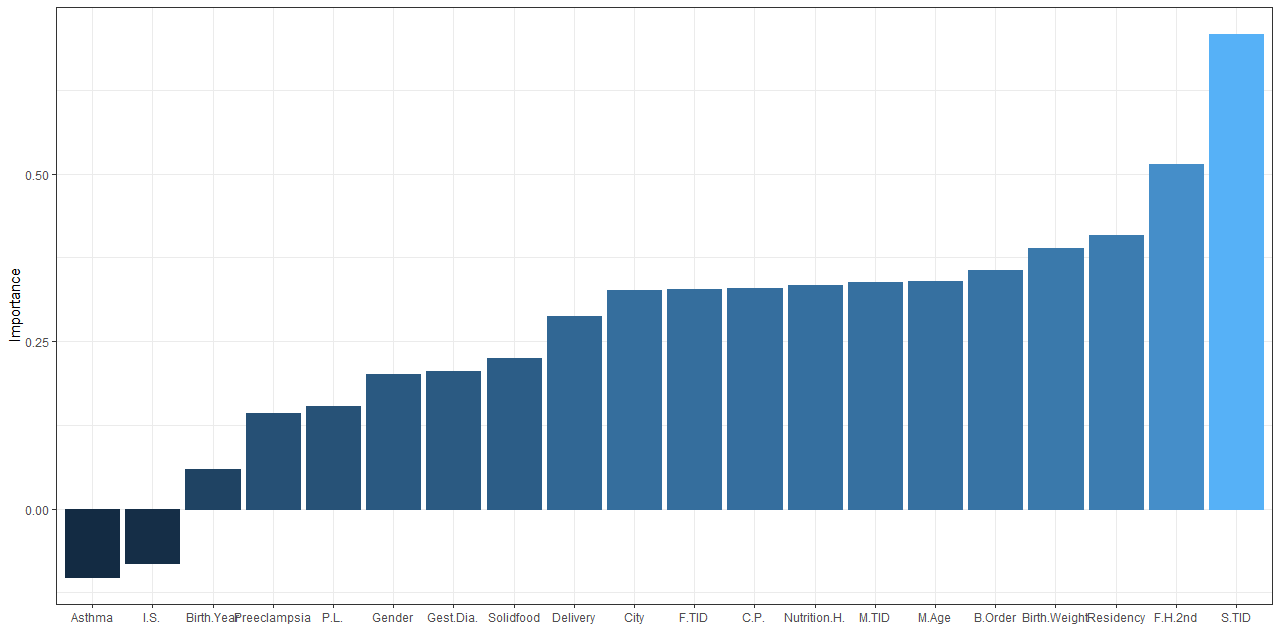

Supplement: S4 Fig — P.L: pregnancy length, F.H.2nd: Family history of T1D with a second-degree relative, I.S.:Income Status, C.P.: Consanguineous Parents, M.Age: Maternal Age, B.Weight: Birth weight, B.Order: Birth Order. (TIF) [file pone.0282426.s005.tif]

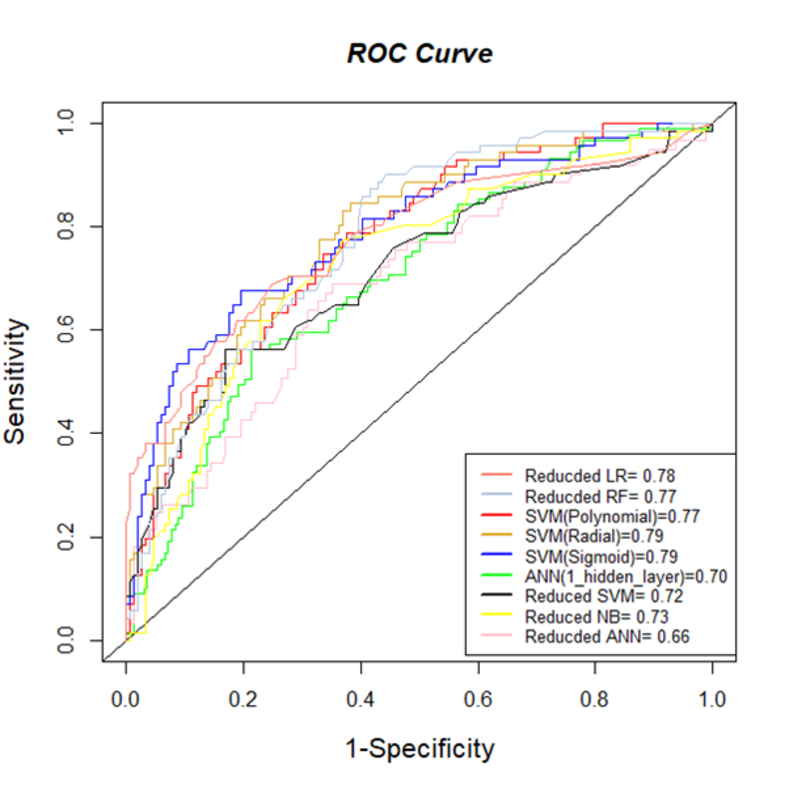

Supplement: S5 Fig — (TIF) [file pone.0282426.s006.tif]
